# Loneliness in adolescence and prescription of psychotropic drugs in adulthood: 23-year longitudinal population-based and registry study

**DOI:** 10.1192/bjo.2024.22

**Published:** 2024-03-11

**Authors:** Rubén Rodríguez-Cano, Karianne Lotre, Tilmann von Soest, Eline Borger Rognli, Jørgen Gustav Bramness

**Affiliations:** Department of Psychology, Norwegian University of Science and Technology (NTNU), Norway; and PROMENTA Research Center, Department of Psychology, University of Oslo, Norway; Institute for Clinical Medicine, UiT – The Arctic University of Norway, Norway; PROMENTA Research Center, Department of Psychology, University of Oslo, Norway; and Norwegian Social Research (NOVA), OsloMet – Oslo Metropolitan University, Norway; Section for Clinical Addiction Research, Division of Mental Health and Addiction, Oslo University Hospital, Norway; Institute for Clinical Medicine, UiT – The Arctic University of Norway, Norway; Department of Alcohol, Tobacco and Drugs, Norwegian Institute of Public Health, Trondheim, Norway; and Norwegian National Advisory Unit on Concurrent Substance Abuse and Mental Health Disorders, Innlandet Hospital Trust, Hamar, Norway

**Keywords:** Loneliness trajectories, drug prescriptions, mental disorders, psychotic disorders, bipolar disorder

## Abstract

**Background:**

The role of adolescent loneliness in adult mental health and prescriptions of psychotropic drugs remains underexplored.

**Aims:**

We aim to determine whether (a) experiencing loneliness in adolescence and (b) changes in loneliness from adolescence to adulthood are prospectively associated with prescriptions for a variety of psychotropic drugs in adulthood.

**Method:**

We used data from a Norwegian population-based sample with 2602 participants, collected across four waves between 1992 and 2006. Loneliness was assessed at each wave, with survey data linked to medicinal drug prescription records from the Norwegian Prescription Database. We identified prescription histories of antipsychotics, mood stabilisers, antidepressants and benzodiazepines from 2007 to 2015, for each participant. We use latent growth curve analyses to model the relationship of adolescent loneliness and loneliness change from adolescence to adulthood, with subsequent psychotropic drugs prescription.

**Results:**

Adolescents with heightened loneliness, and adolescents whose loneliness increased into young adulthood, had a greater likelihood of being prescribed antipsychotics, mood stabilisers and antidepressants in adulthood. These associations remained significant after adjustment for confounders such as sociodemographic characteristics, conduct problems, substance use and mental health problems.

**Conclusions:**

Loneliness in adolescence and its adverse development over a span of 15 years was linked to higher risk of receiving prescriptions for antipsychotics, mood stabilisers and antidepressants later in life. The findings may indicate that loneliness increases the risk for developing psychotic disorders, bipolar disorders and major depression.

Loneliness is a distressing experience that arises when a person perceives a deficiency in social relationships.^1,2^ Loneliness is related to several adverse health conditions, including impaired cognitive function, compromised immune functioning, heightened inflammation, elevated cortisol levels and functional disability.^3,4^ In fact, meta-analytic evidence combining more than 100 prospective studies indicates that loneliness increases the risk of premature death by 22–26%.^5,6^ Remarkably, loneliness has been found to be a greater health risk for premature mortality than well-established risk factors such as smoking,^1,2,7^ underscoring the need to address loneliness as a public health concern.

## Loneliness and mental health

Despite the growing consensus that loneliness is a risk factor for anxiety and depression, the majority of existing research focuses on the adult general population.^8^ There has been little research on how loneliness experienced in adolescence is related to later adult mental health. The two available studies examining this issue suggest that higher levels of loneliness in adolescence may increase the odds of being diagnosed with an anxiety disorder^9^ and receiving antidepressant prescriptions later in life.^10^ However, whether loneliness in adolescence may be also associated with indicators for other mental disorders in adulthood, including severe disorders such as schizophrenia and bipolar disorders, is not known.

Moreover, further investigation is needed on the development of loneliness from adolescence to adulthood, and how changing loneliness patterns are related to the risk of acquiring mental disorders in adulthood. This issue is especially important given the documented increase in loneliness during adolescence and young adulthood,^10,11^ a transitional period when most mental disorders emerge.^12^ The few longitudinal studies that have examined how changes in loneliness are prospectively related with mental health indicate that developmental trajectories with moderate to high levels of loneliness between 9 and 16 years of age were associated with increased symptoms of anxiety in adulthood,^9^ and an increase of loneliness from 13 to 31 years of age predicted subsequent antidepressant use as treatment for depression.^10^ However, how the development of loneliness during adolescence and young adulthood is related to a variety of mental disorders later in life, including severe psychiatric conditions such as schizophrenia and bipolar disorders, remains largely unexplored. By using psychotropic drug prescriptions as proxies for mental disorders, we address this issue in a large-scale epidemiological study.

## The current study

The current study aimed to investigate whether (a) experiencing loneliness in adolescence and (b) the change in loneliness from adolescence to adulthood are prospectively related to receiving psychotropic drug prescriptions in adulthood. Utilising a prospective, population-based sample, we repeatedly assessed loneliness from adolescence to adulthood, linking both the level of loneliness in adolescence and change in loneliness from adolescence to adulthood to national registry data about psychotropic drug prescriptions. We also accounted for relevant confounding factors, such as sociodemographic characteristics, conduct problems, substance use, mental health issues in adolescence and previous prescriptions of psychotropic drugs.

## Method

### Procedure and participants

We recruited participants (*N* = 2602) from the longitudinal Young in Norway Study.^13,14^ The study assessed participants in 1992 (time point 1; mean age 15.24 years, s.d. = 1.96), 1994 (time point 2; mean age 16.93 years, s.d. = 1.75), 1999 (time point 3; mean age 21.84 years, s.d. = 1.76) and 2005–2006 (time point 4; mean age 28.50 years, s.d. = 1.73). The initial sample at the first data collection included 12 655 students in grades 7 to 12 (mean age 15.44 years, s.d. = 1.66). Participants were recruited from 67 representative junior and senior high schools in the country, with a response rate of 97%. At time point 2, about half of the students had completed the 3-year track at the junior or senior high school and had left the school they had been attending at the first data collection. Only students who had completed the questionnaires at school at the second data collection (*n* = 3844) were followed up in time points 3 and 4. The response rates among students eligible for participation were 92% for time point 2, 83% for time point 3 and 82% for time point 4. The overall participation rate, based on all eligible students at time point 1 who still were at their original school at time point 2, was 68% at time point 3 and 67% at time point 4. At time point 4, 2602 participants (56% women) consented to link their questionnaire data to data from the Norwegian Prescription Database (NorPD). Of those, 8.03% (*n* = 209) had missing data on loneliness at time point 1, with 4.77% (*n* = 124), 11.45% and 0.03% (*n* = 1) missing data on loneliness at time points 2, 3 and 4, respectively. The NorPD is a national register containing information about all prescription drugs dispensed at all Norwegian pharmacies to all patients living outside of institutions. As there are no private prescribing or refunding of prescriptions in Norway, the register has complete coverage, with the only exception being drugs given to in-patients. The date the prescription was filled, the anatomical-therapeutic-chemical (ATC) code of the medicine^15^ and number of daily defined doses are recorded in the NorPD for each prescription. We used data for all prescriptions dispensed 1–9 years after time point 4 (1 January 2007 to 31 December 2015). For a detailed information about study participation, see the flow chart in the Supplementary material available at https://doi.org/10.1192/bjo.2024.22.

Attrition analyses were conducted, contrasting participants who had completed time point 1 but dropped out at a later time point with those who remained in the study and consented to register linkage. Results showed that male gender (odds ratio 4.34, 95% CI 1.62–11.64) and being older (odds ratio 1.27, 95% CI 1.03–1.58) were significantly related to dropping out, whereas the remaining covariates and loneliness at time point 1 were not (*P* > 0.05).

The authors assert that all procedures contributing to this work comply with the ethical standards of the relevant national and institutional committees on human experimentation and with the Helsinki Declaration of 1975, as revised in 2008. All procedures involving human participants were approved by the Regional Committees for Medical and Health Research Ethics Norway (reference number: 25462; project name: ‘Young in Norway’).

### Measures

#### Loneliness

We assessed loneliness by using the averaged responses to a five-item scale, with response options ranging from 1 (never) to 4 (often). Four items were based on a short version of the UCLA Loneliness Scale,^16^ including ‘I feel in tune with the people around me’, ‘I can find companionship when I want it’, ‘No one really knows me well’ and ‘People are around me but not with me’. This instrument has reported good reliability and high correlation with the full UCLA Loneliness Scale.^16^ The fifth item, ‘I feel lonely’, assesses loneliness directly. The five-item measure used in this study has been shown to have good face and predictive validity,^10^ and has been used in previous longitudinal studies,^17^ The reliability of the instrument ranged from α = 0.65 to 0.78 in the present study.

#### Prescription of psychotropic drugs

We categorised participants into mutually exclusive groups according to their prescription history of antipsychotics, mood stabilisers, antidepressants and benzodiazepine from 2007 to 2015. We created an additional group that, based on medication types and dosages, were presumed to be prescriptions for conditions other than a mental disorder (mainly nausea, sleep problems or epilepsy). For the additional analyses in the current study, we also create a group comprising any kind of psychotropic drug prescription versus no such prescription. Additionally, we retrieved prescription data for 2004 from the NorPD, the year before time point 4 was completed. NorPD contains all prescriptions filled by all Norwegians living outside of institutions in the period. For the current study, all prescriptions potentially given for mental disorders were included. This included ATC codes N03A E01 (clonazepam), N03A G01 (valproic acid), N03A X09 (lamotrigine), N05* (antipsychotics, anxiolytics and sedative/hypnotics) and N06A* (antidepressants).

If a person received drugs from more than one prescription category during the 9-year follow-up period, we used the following hierarchical priority to allocate that person: (a) mood stabilisers, (b) antipsychotics, (c) antidepressants, (d) benzodiazepines and (e) psychotropic medications presumably prescribed on non-psychiatric indications. The decision rules were based on the fact that certain medications, such as lithium for bipolar disorders, are almost exclusively prescribed for the treatment of a specific psychiatric condition, irrespective of the simultaneous prescription of other psychotropic drugs. Similarly, continuous high-dose prescriptions of antipsychotics indicate psychotic disorders. Such psychotropic drug prescribing was placed at the top of the decision-making hierarchy. In contrast, other psychotropic drugs may be less specific in their indication. For instance, a prescription of antidepressants may imply treatment for unipolar depression, but also bipolar disorder (at least if combined with lithium or other mood stabilisers) or anxiety disorder. Such diversity applies also for some low-dose prescriptions of antipsychotics. In these cases, a broader evaluation was made, considering factors such as co-medication, number of prescriptions and doses prescribed. We also accounted for a few exceptions to the main guidelines to allow for specific pharmacological combinations, such as low-dose prescriptions and instances of single prescriptions. Quetiapine in a lower tablet size than 45 mg per tablet, and prochlorperazine, levomepromazine and chlorprothixene in a lower tablet size than 25 mg per tablet, were not considered antipsychotic medication. For details about the procedure of assigning persons to diagnostic groups based on their prescription history, and information about overlap between prescription categories, see the Supplementary material. Notably, we developed the categorisation criteria without knowledge about loneliness levels.

#### Covariates

##### Sociodemographic characteristics

We assessed participants’ age, gender, country of birth (Norway or other), parental education (from 1 ‘up to 9 years of basic education’ to 5 ‘more than 3 years of university education’) and whether the participant lived with both parents or not at time point 1.

##### Conduct problems

We assessed conduct problems at time point 1 with a 15-item measure. This instrument included most of the DSM-III-R criteria for conduct disorder, and it has been used in previous studies.^18,19^ Response options ranged from 1 (never) to 6 (more than 50 times). We computed the mean scores, and the instrument showed good reliability in our sample (α = 0.75).

##### Substance use

We assessed the use of several substances at time point 1. Alcohol use included the frequency of alcohol intoxication episodes during the past 12 months, assessed by asking how often respondents had drunk so much that they had felt intoxicated. Response options ranged from 1 (never) to 6 (more than 50 times). We assessed cigarette smoking, with response options being non-smoker (0), non-daily smoker (1), smoke <10 cigarettes daily (2) and smoke 10 cigarettes or more daily (3). Cannabis use included whether the participant had consumed cannabis at least once in the previous 12 months.

##### Mental health problems

We assessed symptoms of depression and anxiety at time point 1 with 12 items from the Hopkins Symptom Checklist.^20^ The items referred to mental health problems experienced during the preceding week, and response options ranged from 1 (not bothered at all) to 4 (extremely bothered). This instrument has shown sound psychometric properties in previous studies.^21^ We computed the mean scores, and the instrument showed high internal consistency in the current study (α = 0.85).

### Analyses

We estimated correlations among all the study variables. To address the study aims, we investigated (a) the associations between experiencing loneliness in adolescence (assessed at time point 1) and psychotropic prescriptions in adulthood, and (b) the association between change in loneliness from adolescence to adulthood (assessed from time point 1 to time point 4) and psychotropic prescriptions in adulthood, using latent growth curve models. We tested linear, quadratic and cubic change of loneliness. We evaluated the trajectory of the growth model, using cut-off values of ≥0.95 for the Comparative Fit Index (CFI) and the Tucker–Lewis Index (TLI), ≤0.06 for the root mean square error of approximation (RMSEA) and 0.08 for standardised root mean square residual (SRMR).^22^ A latent growth model with linear change indicated a good model fit: χ²(5) = 64.56, *P* < 0.001, CFI = 0.98, TLI = 0.97, RMSEA = 0.065, SRMR = 0.042. Then, we set the intercept of the models to time point 1, representing the estimated levels of loneliness at time point 1, and we included a linear slope to indicate changes in loneliness from time point 1 to time point 4. We calculated the parameters for the linear slope by dividing the time intervals between the measured points by 10 (to facilitate convergence), with time point 1 at 0.0, time point 2 at 0.2, time point 3 at 0.7 and time point 4 at 1.3. Therefore, a one-unit change in the slope reflected a change in loneliness over one decade.

We used prescription of psychotropic drugs as the dependent variable. First, we ran a hierarchical logistic regression model with a categorical outcome indicating having received any psychotropic drug prescription in adulthood versus not having received any psychotropic prescription. Second, we ran hierarchical multinomial logistic regression analyses, with the categories of antipsychotics, mood stabilisers, antidepressants, benzodiazepines and psychotropic drugs for other indications, all of which were compared with the reference category of not having received any psychotropic prescription. The baseline model included the intercept and linear slope of the growth curve as predictors. In model 1, sociodemographic variables (i.e. age, gender, country of birth, whether the participant lived with both parents or not, and parental education) were controlled for. Model 2 additionally controlled for conduct problems and substance use at time point 1, whereas model 3 additionally controlled for mental health problems at time point 1. Finally, model 4 included the previous models, but excluded those participants who received a psychotropic drug prescription 1 year before time point 4, in 2004 *(n* = 134). The results were presented as odds ratios with 95% confidence intervals.

To account for potential non-normality in the analyses, we used a robust maximum likelihood estimator.^23^ We also accounted for the potential non-independence of observations resulting from school clusters (*n* = 67) in the estimation of parameters by using a maximisation-weighted log-likelihood function, with standard error estimates obtained with a sandwich estimator. We used full information maximum likelihood to account for missing data, which is considered to be a state-of-the-art procedure for handling missingness.^23,24^ We conducted all analyses with software Mplus for Windows version 8.5 (Muthén & Muthén, https://www.statmodel.com).^23^

## Results

### Sample characteristics and descriptive statistics

Descriptive information is presented in Table 1. Most of the participants (97%) were born in Norway and were living with both parents at time point 1 (70%). Most had not received any psychotropic drug prescriptions between 2007 and 2015 (*n* = 2114, 81%), whereas 488 (19%) participants had received at least one such prescription. Of all participants, 301 individuals (12%) had received prescriptions from only one category of medication, and 187 (7%) individuals had received prescriptions from two or more categories. Of all participants, 33 individuals (1.3%) were classified as recipients of antipsychotics, 36 individuals (1.4%) as recipients of mood stabilisers, 233 individuals (9.0%) as recipients of antidepressants, 102 individuals (3.9%) as recipients of benzodiazepines and 84 individuals (3.2%) were prescribed psychotropic drugs presumably on non-psychiatric indications. Intercorrelations among all study variables are displayed in Table 2. We found that loneliness was moderately to strongly correlated between time points (*r* = 0.35–0.58). Adolescents’ mental health problems at time point 1 were also positively related with loneliness at all time points (*r_t1_* = 0.41, *r_t2_* = 0.30, *r_t3_* = 0.25, *r_t4_* = 0.21).

**Table 1:** Descriptive statistics by psychotropic prescription

Variable		Overall (*N* = 2602)	Antipsychotics (*n* = 33)	Mood stabilisers (*n* = 36)	Antidepressants (*n* = 233)	Benzodiazepines (*n* = 102)	Psychotropic drugs on other indications (*n* = 84)	Non-psychotropic drugs (*n* = 2114)
Loneliness time point 1	Mean (s.d.)	1.86 (0.54)	2.11 (0.45)	1.86 (0.62)	1.98 (0.59)	1.81 (0.55)	1.98 (0.50)	1.84 (0.53)
Loneliness time point 2	Mean (s.d.)	1.82 (0.56)	1.84 (0.56)	1.79 (0.61)	1.96 (0.62)	1.71 (0.48)	1.83 (0.57)	1.80 (0.55)
Loneliness time point 3	Mean (s.d.)	1.80 (0.50)	2.01 (0.49)	1.97 (0.47)	1.99 (0.54)	1.82 (0.56)	1.77 (0.47)	1.78 (0.49)
Loneliness time point 4	Mean (s.d.)	1.78 (0.50)	2.30 (0.63)	2.19 (0.55)	2.01 (0.56)	1.78 (0.54)	1.77 (0.50)	1.74 (0.47)
Age time point 1	Mean (s.d.)	15.24 (1.96)	14.88 (3.18)	15.14 (3.04)	15.28 (2.15)	15.37 (1.92)	15.37 (2.41)	15.24 (1.87)
Gender								
Male	*n* (%)	1145 (44%)	18 (55%)	11 (31%)	79 (34%)	33 (32%)	27 (32%)	977 (46%)
Female	*n* (%)	1457 (56%)	15 (45%)	25 (69%)	154 (66%)	69 (68%)	57 (68%)	1137 (54%)
Born in Norway								
No	*n* (%)	74 (3.0%)	2 (6.9%)	2 (5.9%)	7 (3.2%)	3 (3.1%)	1 (1.3%)	59 (3.0%)
Yes	*n* (%)	2369 (97%)	27 (93%)	32 (94%)	214 (97%)	94 (97%)	77 (99%)	1925 (97%)
Parental education	Mean (s.d.)	3.38 (1.12)	3.08 (1.13)	3.33 (1.18)	3.16 (1.07)	3.33 (1.12)	3.21 (1.10)	3.42 (1.12)
Living with only one parent								
No	*n* (%)	1736 (69%)	20 (65%)	24 (71%)	130 (58%)	56 (55%)	56 (70%)	1450 (71%)
Yes	*n* (%)	765 (31%)	11 (35%)	10 (29%)	96 (42%)	45 (45%)	24 (30%)	579 (29%)
Alcohol use time point 1	Mean (s.d.)	1.91 (1.43)	1.54 (1.10)	1.57 (1.25)	2.11 (1.46)	2.10 (1.61)	2.09 (1.47)	1.88 (1.42)
Smoking time point 1	Mean (s.d.)	0.36 (0.83)	0.30 (0.84)	0.47 (1.02)	0.55 (1.02)	0.65 (1.07)	0.35 (0.73)	0.33 (0.79)
Cannabis use time point 1								
No	*n* (%)	2306 (97%)	25 (89%)	26 (87%)	208 (97%)	92 (95%)	73 (97%)	1882 (97%)
Yes	*n* (%)	78 (3.3%)	3 (11%)	4 (13%)	7 (3.3%)	5 (5.2%)	2 (2.7%)	57 (2.9%)
Conduct problems time point 1	Mean (s.d.)	1.36 (0.39)	1.36 (0.35)	1.37 (0.46)	1.41 (0.40)	1.45 (0.53)	1.41 (0.45)	1.34 (0.37)
Mental health problems time point 1	Mean (s.d.)	1.59 (0.46)	1.70 (0.55)	1.65 (0.48)	1.80 (0.53)	1.64 (0.44)	1.65 (0.45)	1.57 (0.45)
Psychotropic drug prescription in 2004								
No	*n* (%)	2468 (95%)	21 (64%)	17 (47%)	191 (82%)	92 (90%)	73 (87%)	2074 (98%)
Yes	*n* (%)	134 (5.1%)	12 (36%)	19 (53%)	42 (18%)	10 (9.8%)	11 (13%)	40 (1.9%)

Time point 1 corresponds to the year 1992, time point 2 corresponds to the year 1994, time point 3 corresponds to the year 1999 and time point 4 corresponds to the year 2005.

**Table 2:** Intercorrelations among all study variables (*N* = 2602)

Variable	1	2	3	4	5	6	7	8	9	10	11	12	13	14
1. Loneliness time point 1														
2. Loneliness time point 2	0.58**													
3. Loneliness time point 3	0.38**	0.48**												
4. Loneliness time point 4	0.35**	0.42**	0.55**											
5. Age time point 1	−0.02	−0.05*	−0.03	−0.03										
6. Female	0.05*	0.06**	0.05*	0.05*	0.04									
7. Born in Norway	−0.06**	−0.03	−0.04	−0.06**	−0.02	−0.01								
8. Parental education	−0.06*	−0.04	−0.07**	−0.04	0.02	−0.00	−0.05*							
9. Live with only one parent	0.04	0.04*	0.07**	0.09**	0.03	0.02	−0.09**	−0.02						
10. Alcohol use time point 1	0.07**	−0.06**	−0.05**	−0.02**	0.47**	0.02	0.04	0.04	0.13**					
11. Smoking time point 1	−0.01	0.02	0.03	0.04	0.29**	0.07**	−0.02	−0.01	0.16**	0.51**				
12 Cannabis use time point 1	0.02	0.01	0.02	0.05*	0.16**	−0.02	−0.04	0.07**	0.08**	0.32**	0.29**			
13. Conduct problems time point 1	0.00	0.00	0.00	0.03	0.17**	−0.18**	−0.01	0.02	0.12**	0.48**	0.38**	0.30**		
14. Mental health problems time point 1	0.41**	0.30**	0.25**	0.21**	0.11**	0.24**	−0.06**	−0.00	0.06**	0.14**	0.20**	0.08**	0.13**	
15. Have a psychotropic drug prescription in 2004	0.05*	0.04	0.04	0.15**	0.02	0.07**	−0.01	−0.03	0.06**	0.03	0.05*	0.09**	0.06**	0.06**

Time point 1 corresponds to the year 1992, time point 2 corresponds to the year 1994, time point 3 corresponds to the year 1999 and time point 4 corresponds to the year 2005.

* *P* < 0.05, **P < 0.01.

### Loneliness and psychotropic prescriptions

Results from hierarchical logistic regression models predicting any type of psychotropic drug prescription are displayed in Table 3. Results show that significant associations between high loneliness levels in adolescents and adverse change in loneliness in models both with and without control for covariates. More specifically, a high level of loneliness in adolescence (odds ratio 1.83, 95% CI 1.33–2.52) and a greater increase in loneliness from adolescence to adulthood (odds ratio 3.09, 95% CI 2.15–4.44) increased the risk of receiving any psychiatric medication in adulthood, when controlling for all covariates in model 3. Moreover, the relationships remained significant even when excluding all participants who received prescriptions in 2004, in model 4.

**Table 3:** Results of multinomial logistic regressions of loneliness as predictor and prescription of psychotropic drugs as outcome

	Any psychotropic drug prescription		Antipsychotics		Mood stabilisers		Antidepressants		Benzodiazepines		Psychotropic drugs on other indications	
	Odds ratio	95% CI	Odds ratio	95% CI	Odds ratio	95% CI	Odds ratio	95% CI	Odds ratio	95% CI	Odds ratio	95% CI
Baseline model: unadjusted associations												
Loneliness at time point 1	2.17	2.02–3.65	7.40	3.60–15.20	3.65	1.42–9.42	4.19	2.77–6.33	0.95	0.50–1.83	1.45	0.81–2.60
Loneliness change (time point 1 to time point 4)	3.51	2.36–5.13	20.92	4.17–105.01	24.33	5.86–100.97	4.18	2.28–7.66	1.97	0.93–4.17	0.77	0.32–1.86
Model 1: adjustment for sociodemographic characteristics												
Loneliness at time point 1	2.47	1.87–3.27	6.65	3.20–13.74	3.40	1.28–8.72	3.77	2.53–5.63	0.84	0.44–1.58	1.40	0.79–2.48
Loneliness change (time point 1 to time point 4)	3.28	2.27–4.73	21.14	4.40–101.65	17.91	5.11–62.73	3.86	5.11–62.74	1.72	0.85–3.50	0.74	0.31–1.77
Model 2: additional adjustment for conduct problems and substance use												
Loneliness at time point 1	2.38	1.79–3.15	6.08	2.86–12.93	2.63	0.99–6.99	3.69	2.50–5.46	0.77	0.41–1.46	1.41	0.80–2.48
Loneliness change (time point 1 to time point 4)	3.04	2.11–4.38	17.14	3.82–76.91	18.06	4.49–72.70	3.47	2.05–5.94	1.61	0.78–3.33	0.75	0.31–1.82
Model 3: additional adjustment for mental distress												
Loneliness at time point 1	1.83	1.33–2.52	4.21	1.66–10.66	2.15	0.75–6.14	2.54	1.63–3.97	0.66	0.31–1.42	1.42	0.77–2.60
Loneliness change (time point 1 to time point 4)	3.09	2.15–4.44	16.85	3.92–72.49	17.64	4.45–69.94	3.66	2.11–6.35	1.62	0.80–3.29	0.76	0.32–1.85
Model 4: additional adjustment for use of psychotropic drugs in 2004												
Loneliness at time point 1	1.76	1.22–2.54	3.91	1.26–12.15	5.98	1.82–19.72	2.41	1.44–4.04	0.75	0.38–1.47	1.13	0.59–2.18
Loneliness change (time point 1 time point 4)	2.45	1.76–3.41	11.22	1.90–66.07	8.78	1.80–42.89	3.52	2.04–6.08	1.38	0.71–2.68	0.66	0.26–1.66

Reference category for multinomial logistic regression is no psychotropic drug prescription. Time point 1 corresponds to the year 1992, time point 2 corresponds to the year 1994, time point 3 corresponds to the year 1999 and time point 4 corresponds to the year 2005. Model 1: control variables were age, gender, being born in Norway, living with both biological parents and parental education. Model 2: control variables were model 1 controls and conduct problems at time point 1 and substance use at time point 1 (alcohol, smoking cigarettes, cannabis). Model 3: control variables were model 2 controls and mental health problems at time point 1. Model 4: control variables were model 3 controls, with participants who obtained prescriptions for psychotropic drugs in 2004 (*n* = 134) removed from the analyses. Analyses were run with 2468 participants.

Next, we examined how loneliness was related to prescription of specific types of psychotropic drugs. Table 3 shows that higher levels of loneliness during adolescence were associated with higher likelihoods of filling prescriptions for antipsychotics, mood stabilisers and antidepressants. In particular, after controlling for sociodemographic characteristics, conduct problems, substance use and other mental health issues, as well as excluding individuals who had received a psychotropic drug prescription in 2004, the relationships remained stable and robust for receiving prescriptions of antipsychotics (odds ratio 3.91, 95% CI 1.26–12.15), mood stabilisers (odds ratio 5.98, 95% CI 1.82–19.72) and antidepressants (odds ratio 2.41, 95% CI 1.44–4.04), when compared with those who did not receive any psychotropic prescription in adulthood. However, adolescent loneliness levels did not predict the prescription of benzodiazepine or psychotropic medications for other indications.

A greater linear increase in loneliness from adolescence to adulthood was associated with a heightened likelihood of receiving several specific types of prescriptions in adulthood. After controlling for sociodemographic characteristics, conduct problems, substance use and other mental health issues, as well as excluding individuals who had received a psychotropic drug prescription in 2004, the relationships remained stable for receiving prescriptions of antipsychotics (odds ratio 11.22, 95% CI 1.90–66.07), mood stabilisers (odds ratio 8.78, 95% CI 1.80–42.89) and antidepressants (odds ratio 3.52, 95% CI 2.04–6.08), compared with those without prescriptions in adulthood.

**Fig. 1:**
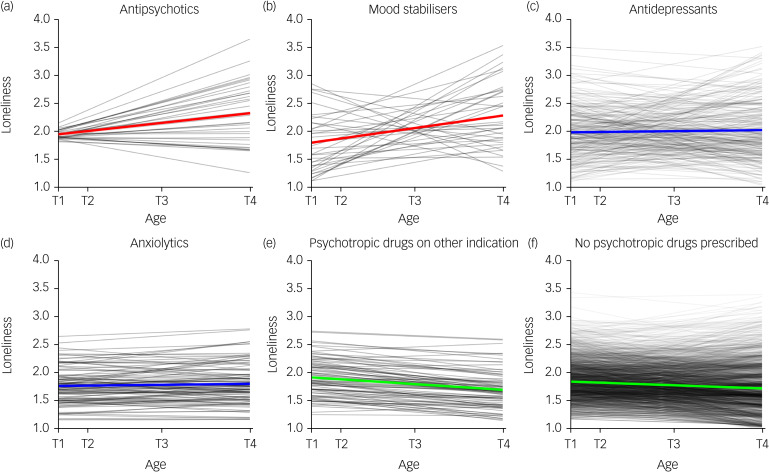
Predictive values for the linear latent growth curve model by psychotropic drug prescription. Red lines indicate a statistically significant linear increase of loneliness. Blue lines indicate a non-significant change of loneliness. Green lines indicate a statistically significant linear reduction in loneliness. Age at time point 1: mean 15.24 years, s.d. = 1.96; age at time point 2: mean 16.93 years, s.d. = 1.75; age at time point 3: mean 21.84 years, s.d. = 1.76; age at time point 4: mean 28.50 years.

Figure 1 displays the loneliness change for each prescription group. Those who received drugs for psychotic disorders (unstandardised coefficient *β* = 0.62, *P* = 0.023) and bipolar disorder (*β* = 0.49, *P* = 0.017) during adulthood experienced a significant increase in loneliness from adolescence to adulthood. Those with a prescription for psychotropic medications for other indications (*β* = −0.66, *P* = 0.004) and those without prescriptions *(β* = −0.23, *P* **<** 0.001) demonstrated a decline in loneliness across adolescence and adulthood.

## Discussion

There is limited research on how adolescent loneliness and its progression into adulthood may contribute to subsequent mental disorders. Our study aimed to fill this gap by investigating the relationship between self-reported loneliness and the prescription of psychotropic drugs. We utilised a population-based longitudinal data-set alongside national registry data for psychotropic prescriptions. We found that greater loneliness in adolescence and a greater increase in loneliness from early adolescence to adulthood were associated with future risk of receiving any psychotropic prescriptions in general, as well as prescriptions for antipsychotics, mood stabilisers and antidepressants during adulthood. Notably, these associations remained significant after controlling for sociodemographic factors, conduct problems, substance use, mental health issues and previous psychotropic drug prescriptions. In contrast, loneliness levels and changes from adolescence to adulthood did not predict the prescription of benzodiazepines or psychotropic drugs on other indications.

Our results of loneliness predicting prescriptions of mood stabilisers and antidepressants are consistent with previous research linking loneliness in early adulthood with the subsequent onset of bipolar disorder and depression during adulthood.^8^ We also found that loneliness in adolescence is related to prescriptions of antipsychotics during adulthood. Previous meta-analytic evidence suggested that there is a positive relationship between loneliness and psychotic symptoms among people with psychotic disorders.^25^ However, most of the previous evidence were cross-sectional, did not distinguish between psychotic and bipolar disorders, and included only adults.^25^ In contrast, our results expand the previous literature suggesting a long-term prospective association between experiencing loneliness in adolescence and subsequent prescriptions of antipsychotics and mood stabilisers. Importantly, we assessed loneliness during adolescence, which is usually the period that precedes the onset of schizophrenia spectrum and bipolar disorders.^12^ Although we cannot conclude that there is a causal link between loneliness and later severe mental disorders, our results indicate that early adolescent loneliness precedes the prescription of drugs commonly used to treat psychotic and bipolar disorders during adulthood.

Loneliness could be a consequence of prodromal psychotic symptoms. For instance, difficulties in social interactions, impaired social skills and social anhedonia^26,27^ may lead to diminished social networks and increased feelings of loneliness. On the other hand, loneliness could also be a risk factor for severe mental disorders. Cognitive theories of psychotic symptomatology posit that a poor self-concept and low self-esteem could increase firmly held cognitive distortions about one's self (such as viewing oneself as a distinct individual) and others (such as perceiving others as hostile).^28^ In addition, diminished social self-efficacy may negatively affect individuals’ self-assessment of their social skills, potentially enhancing feelings of loneliness. This implies that individuals with psychosis who concurrently experience loneliness may possess more complex social cognition relative to their healthy counterparts.^29^ Therefore, loneliness could serve as a mechanism that exacerbates already existing prodromal psychotic symptoms. Future studies should investigate whether loneliness is a mechanism by which prodromal psychotic symptoms during adolescence develop into established psychotic symptoms during adulthood.

Contrary to some studies,^8,9^ but in agreement with others,^30^ we did not find a relationship between loneliness and benzodiazepines, being indicative of anxiety disorder. Common underlying genetic and environmental influences related to loneliness and depression might explain why loneliness increases later depression, but not anxiety.^31^ In addition, some methodological aspects of our study might explain these differences. For example, although we used an objective measure for prescriptions in the form of a national prescription registry (i.e. the NorPD), the benzodiazepine category was at the bottom of the decisional hierarchy, implying a more uncertain association between prescriptions and mental disorders compared with the prescription categories higher up in the hierarchy. Furthermore, modern day antidepressants are frequently considered as the primary treatment option for severe anxiety.^32,33^ Thus, some cases of anxiety, particularly more severe ones, might have been inadvertently classified under the antidepressant category. Therefore, future studies should aim to more closely examine the longitudinal relationship between loneliness and specific types of anxiety disorders.

In terms of developmental change of loneliness from adolescence to adulthood, our findings align with previous research indicating that an increase of loneliness predicts depressive disorders and antidepressant prescriptions.^9,10^ Previous research focusing uniquely on adolescence found that experiencing chronic loneliness during this period increases the psychopathological symptomatology, such as risk of suicidality, self-harm and suicidal ideation by 16 years of age.^34^ We extend the current literature by demonstrating that a persistent experience of loneliness from adolescence to adulthood is associated with an increased likelihood of prescriptions of antipsychotics or mood stabilisers in adulthood, indicative of schizophrenia or bipolar disorders, respectively. Prior studies have shown that the development of a psychotic disorder might impair social functioning.^35^ Additionally, when a psychotic disorder is already present, increased utilisation of antipsychotics has been linked to a greater decline in social functioning.^35^ Moreover, experiencing higher daily levels of loneliness has been linked to diminished social functioning in psychosis.^36^ Thus, it is plausible that prodromal social symptoms observed in individuals with schizophrenia spectrum disorders may contribute to an escalation of loneliness. This could potentially exacerbate the manifestation of symptoms and social impairment, leading to increased hospital admissions and the utilisation of clinical resources.

The results of our investigation must be interpreted in light of some limitations. Although we categorised participants into mutually exclusive groups according to having received psychotropic prescriptions as a proxy for underlying mental disorders, it does not perfectly mirror the underlying mental disorders. For example, mood stabilisers could be used in some cases as an adjunct medication to psychotic disorders,^37^ and antidepressants are also used for treating anxiety disorders.^32,33^ However, our categorisation was built with rational clinical and pharmacological deliberations, forming a robust hierarchy of the mental disorders’ severity. Our examination of the association between loneliness and any psychotropic drug prescriptions revealed a similar pattern of results to those observed specifically with antipsychotics, mood stabilisers and antidepressants. These results emphasise the broad role of loneliness and loneliness development from adolescence to adulthood in the prescription of psychotropic drugs. It is important to note that we lack information on medication adherence; however, we use prescriptions as a proxy of underlying mental disorder, and did not aim to investigate treatment outcome. In addition, our results are limited by potential bias from attrition or non-consent to linkage to prescription data. Moreover, although our use of longitudinal data allowed for observing changes in loneliness over time, the findings do not conclusively establish causal relationships. Lastly, the data, exclusive to Norway's general population, limit the generalisability of our findings, underscoring the need for replication in varied sociocultural contexts.

In conclusion, this study extends findings from previous studies. We used one of the broadest age ranges for the measurement of loneliness and mental health outcome (23 years), including an age span form adolescence to adulthood, an objective measure of prescription of psychotropic drug medications (data from national patient registries) and indicators of a broad range of mental disorders (including psychotic and bipolar disorders). Nevertheless, to better comprehend the relationship between loneliness and mental disorders, we need more research on the potential role of loneliness in the aetiology of mental disorders. The study results provide first indications that early monitoring of loneliness and interventions aimed at reducing loneliness (e.g. social skill training or structural interventions such as change of school) may benefit those at risk of developing mental disorders, particularly those who experience loneliness over an extended period of time in adolescence and early adulthood.

## Supporting information

Rodríguez-Cano et al. supplementary material Rodríguez-Cano et al. supplementary material

## Data Availability

Data are not publicly available due to sensitivity reasons and ethical requirements.
