# Supplementary material for: Loneliness in adolescence and prescription of psychotropic drugs in adulthood: 23-year longitudinal population-based and registry study
Source: BJPsych Open. 2024 Mar 11;10(2):e61. doi: 10.1192/bjo.2024.22 (PMC10951847; doi:10.1192/bjo.2024.22)
Supplement: Rodríguez-Cano et al. supplementary material [file S205647242400022Xsup001.docx]

**SUPPLEMENT**

**DECISION RULES FOR CATEGORIZATION OF PSYCHOTROPIC DRUGS BASED ON ATC-CODES**

By reviewing all psychotropic drug medication for each participant individually, we aimed to establish whether the medication the person received over the observational period of 9 years (2017-2015) indicated whether they mostly received antipsychotics, mood stabilizers, antidepressants, or anxiolytics, or whether the drugs probably were given on another indication than a mental disorder. The authors (EBR and JGB) performed several iterations of reviewing each case and establishing overriding rules that could applied more generally.

**Hierarchy for categorization**

It is immanent in the rules below, but in this simplified summary, we indicate the hierarchy that was used to decide whether a person received their psychotropic drugs for a specific condition:

- Mood stabilizers were prioritized over other psychotropic drugs.
- For the remaining, antipsychotics in antipsychotic doses were prioritized over antidepressants, anxiolytics and drugs on other indications.
- For the remaining, antidepressants were prioritized over anxiolytics and drugs on other indications.
- For the remaining, anxiolytics were prioritized over drugs on other indications.
- Psychotropic drugs on other indications were coded if the person had received psychotropic medicines, but according to the rules none of the above categories could be applied.

**Decision rules for ascribing prescription category**

1. The following drugs were coded as “benzodiazepine anxiolytics”: NO5BA01 (diazepam), NO5BA04 (oxazepam), NO5BA12 (alprazolam) and NO3AE01 (clonazepam). The exception were
   - if the diazepam was given as rectal medication, in which case it was considered as medication for epilepsy and therefore coded “psychotropic drugs on other indications”. If diazepam as rectal medication was given together with other psychotropic drugs, the person was categorized into “mood stabilizers”, “antipsychotics”, “antidepressants” or “benzodiazepine anxiolytics” if indication for such categorization were present.
2. All N06A* were coded as “antidepressants”, with the following exceptions:
   - N06AX03 (mianserin), N06AA09 (amitriptyline) and N06X11 (mirtazapine) **only** given in 10 mg tablet size (low-dose antidepressants), these were categorized as “psychotropic drugs on other indications”, or as “benzodiazepine anxiolytics” if indication for such categorization were present. N06AX03 (mianserin), N06AA09 (amitriptyline) and N06X11 (mirtazapine) given in larger tablet size than 10 mg were categorized as “antidepressants” (also those who for instance are prescribed 30 + 10 mg).
   - Antidepressants were prioritized over anxiolytics.
3. All N05A* were coded as “antipsychotics”. These were prioritized over “antidepressants” and “benzodiazepine anxiolytics”, with the following exceptions:
   - If the person received only one prescription of the antipsychotic drugs, In that case, “antidepressants” or “benzodiazepine anxiolytics” were coded, or if there was no indication to do so, “psychotropic drugs on other indication” was coded.
   - If the antipsychotic drug was N05AN01 (lithium), “mood stabilizers” was coded. At least one prescription of N05AN01 (lithium), or more than one prescription of N03AF01 (carbamazepine), N03AG01 (valproic acid) or N03AX09 (lamotrigine) was prioritized over antipsychotics, antidepressants and anxiolytics. See however rule 5 for specifications of N03AF01 (carbamazepine).
   - If the antipsychotic drug was:
     1. N05AH04 (quetiapine) in lower tablet size than 45 mg/tablet
     2. N05AB04 (prochlorperazine) lower than 25 mg
     3. N05AA02 (levomepromazine) lower than 25 mg
     4. N05AF03 (chlorprothixene) lower than 25 mg

in which case antidepressants or anxiolytics were prioritized. If only one or more of these (i-vi) were prescribed in low doses, and no other psychotropic drug is prescribed, the category «psychotropic drugs on other indications» was used.

1. If N05AN01 (lithium), N03AF01 (carbamazepine), N03AG01 (valproic acid) or N03AX09 (lamotrigine) were given together with antipsychotics, antidepressants or anxiolytics, the category was “mood stabilizers” if there were more than one prescription of N03AF01 (carbamazepine), N03AG01 (valproic acid) or N03AX09 (lamotrigine), and at least one prescription of N05AN01 (lithium). N05AN01 (lithium), and N03AF01 (carbamazepine), N03AG01 (valproic acid) or N03AX09 (lamotrigine) with more than one prescription, were prioritized over antipsychotics, antipsychotics and anxiolytics. Exceptions:
   - When low dose antidepressants were prescribed together with N03AF01 (carbamazepine) we assumed it to be medication for epilepsy and sleep difficulties, and the person was categorized as “psychotropic drugs on other indications”.
   - N03AF01 (carbamazepine) prescribed together with N05BA01 (diazepam) as rectal medication were assumed to be cases of epilepsy, and categorized as “psychotropic drugs on other indications”.
   - N03AF01 (carbamazepine) together with low dose antidepressants and N05BA01 (diazepam) as rectal medication, and no other types of psychotropic prescriptions, were categorized as “psychotropic drugs on other indications”.
2. Persons who were only prescribed N03AF01 (carbamazepine), N03AG01 (valproic acid) or N03AX09 (lamotrigine), or a combination of these, and no other psychotropic drugs, were categorized as “psychotropic drugs on other indications”. These were assumed to be cases of epilepsy.

- It is evident from the rules above, but we specify here: “Mood stabilizers” were coded in cases of
  - At least one prescription of N05AN01 (lithium)
  - A combination of N03AF01 (carbamazepine), N03AG01 (valproic acid) or N03AX09 (lamotrigine) with anxiolytics, antidepressants or antipsychotics, if more than one prescription of mood stabilizer was given.
  - N03AG01 (carbamazepine) and/or N03AX09 (lamotrigine) with more than one prescription, and no other types of psychotropic drugs.


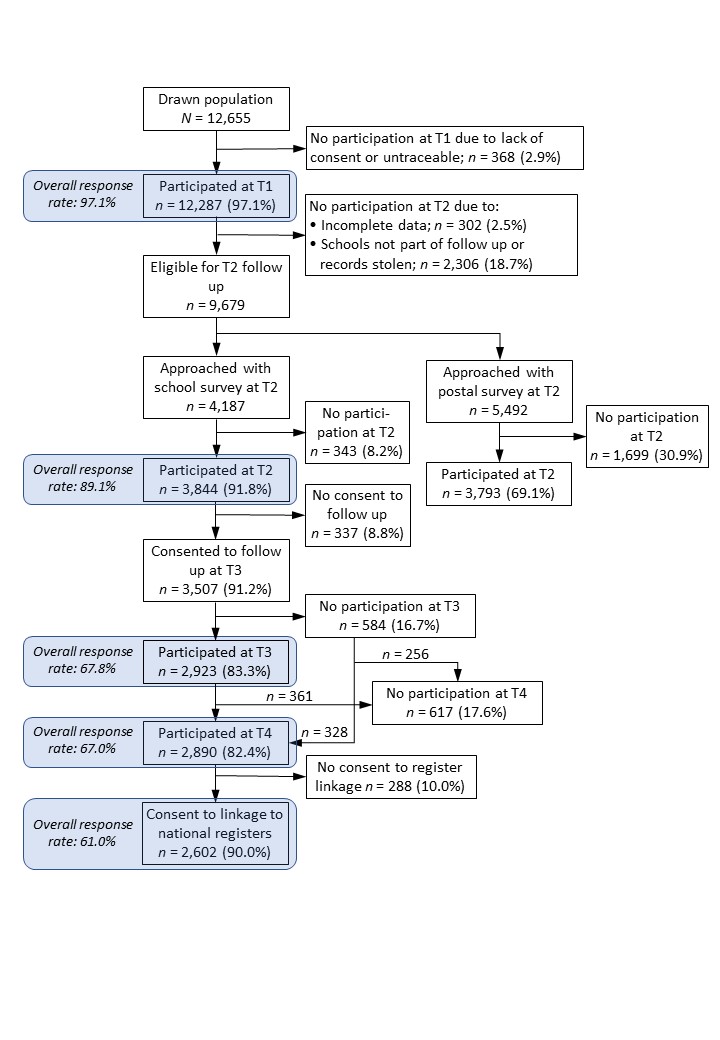
**SUPPLEMENTARY PARTICIPANTS SELECTION FLOW CHART**

**SUPPLEMENTARY TABLES**

**Table S1.** Number and percentage of persons who received specific classes of psychotropic drugs, according to whether they received prescriptions for other classes of psychotropic drugs

| Type of prescriptions received | Did not receive pre­scriptions for other classes of psychotropic drug | Did also receive prescriptions for... | | | | Total |
| --- | --- | --- | --- | --- | --- | --- |
|  |  | Antipsychotics | Mood stabilizers | Antidepressants | Anxiolytics |  |
|  | n (%) | n (%) | n (%) | n (%) | n (%) | n (%) |
| Antipsychotics | 39 (30.5%) |  | 23 (18.0%) | 81 (63.3%) | 52 (40.6%) | 128 (100.0%) |
| Mood stabilizers | 14 (25.5%) | 23 (41.8%) |  | 32 (58.2%) | 25 (45.5%) | 55 (100.0%) |
| Antidepressants | 156 (47.3%) | 81 (24.6%) | 32 (9.7%) |  | 132 (40.0%) | 330 (100.0%) |
| Anxiolytics | 92 (39.3%) | 52 (22.2%) | 25 (10.6%) | 132 (56.4%) |  | 234 (100.0%) |

**Table S2.** Number and percentage of persons who received prescription of specific classes of psychotropic drugs according to the number of different psychotropic drug classes prescribed to them

| Number of classes of psychotropic drugs prescribed | Antipsychotics | Mood stabilizers | Antidepressants | Benzodiazepine  Anxiolytics | Any psychotropic drug |
| --- | --- | --- | --- | --- | --- |
|  | n (%) | n (%) | n (%) | n (%) | n (%) |
| 1 | 39 (30.5%) | 14 (25.5%) | 156 (47.3%) | 92 (39.3%) | 301 (61.7%) |
| 2 | 36 (28.1%) | 16 (29.1%) | 117 (35.5%) | 89 (38.0%) | 129 (26.4%) |
| 3 | 39 (30.5%) | 11 (20.0%) | 43 (13.0%) | 39 (16.7%) | 44 (9.0%) |
| 4 | 14 (10.9%) | 14 (25.5%) | 14 (4.2%) | 14 (6.0%) | 14 (2.9%) |
| Total | 128 (100.0%) | 55 (100.0%) | 330 (100.0%) | 234 (100.0%) | 488 (100.0%) |
